# Larval habitat characteristics of the main malaria vectors in the most endemic regions of Colombia: potential implications for larval control

**DOI:** 10.1186/s12936-015-1002-y

**Published:** 2015-12-01

**Authors:** Marcela Conde, Paula X. Pareja, Lorena I. Orjuela, Martha L. Ahumada, Sebastian Durán, Jennifer A. Jara, Braian A. Cañon, Pilar Pérez, John C. Beier, Socrates Herrera, Martha L. Quiñones

**Affiliations:** Departamento de Salud Pública, Facultad de Medicina, Universidad Nacional de Colombia, Bogotá, DC Colombia; Grupo de Entomología Instituto Nacional de Salud, Bogotá, DC Colombia; Departamento de Estadistica, Facultad de Ciencias, Universidad Nacional de Colombia, Bogota, DC Colombia; Laboratorio de Salud Pùblica, Instituto Departamental de Salud de Nariño, Nariño, Colombia; Department of Public Health Sciences, University of Miami Miller School of Medicine, Miami, FL USA; Caucaseco Scientific Research Centre/Immunology Institute, Universidad Del Valle, Cali, Colombia

**Keywords:** *Anopheles*, Larval habitats, Malaria, Latin America

## Abstract

**Background:**

Malaria incidence has recently decreased globally and, as malaria elimination is envisioned as a possibility by the health authorities, guidance is needed to strengthen malaria control strategies. Larval source treatment, which could complement routine vector control strategies, requires knowledge regarding the *Anopheles* larval habitats.

**Methods:**

A cross-sectional study was conducted in three of the most malaria-endemic regions in Colombia. A total of 1116 potential larval habitats in 70 villages were sampled in three states located in western Colombia: Cordoba, Valle del Cauca and Nariño.

**Results:**

Overall, 17.5 % (195) of the potential larval habitats were found positive for different *Anopheles* species. A total of 1683 larvae were identified belonging to seven species: *Anophelesalbimanus*, *Anopheles calderoni*, *Anophelesdarlingi*, *Anophelesneomaculipalpus*, *Anophelesnuneztovaris.l.*, *Anophelespseudopunctipennis*, and *Anophelestriannulatus*. The most widely distributed species was *An. nuneztovari s.l.*, which was found mainly in human-made fishponds in Cordoba and temporary puddles in Valle del Cauca. *Anophelesalbimanus* and *An. calderoni* were associated with human-made wells or excavation sites in Nariño. Cordoba displayed the greatest *Anopheles* species diversity with a total of six species (Shannon diversity index H′: 1.063). Although Valle del Cauca had four species, one more than Nariño, the diversity was lower because only one species predominated, *An. nuneztovari s.l.* The larval habitats with the highest Shannon diversity index were lagoons (H′: 1.079) and fishponds (H′: 1.009) in Cordoba, excavation sites in Nariño (H′: 0.620) and puddles in Valle del Cauca (H′: 0.764).

**Conclusions:**

This study provides important information regarding the larval habitats of the main malaria vectors in the most malaria-endemic regions of Colombia, which will be useful in guiding larval control operations.

## Background

Between 2000 and 2013 global malaria incidence and mortality rates decreased 30 and 47 %, respectively, based on the estimated number of cases for every 1000 persons [1]. If this trend continues, it is estimated that global malaria incidence may decrease up to 35 % by the end of 2015. In the Americas, malaria cases have decreased from 1.2 million cases in 2000 to 427,000 cases in 2013 (64 %) and mortality decreased by 79 % for the same period. Based on the incidence of cases reported for those years, it is predicted that by the end of 2015 a total of 15 endemic countries in the region will have reached a reduction of 75 % and three other countries a reduction between 50 and 75 % [1].

The majority of countries in Latin America are moving toward malaria elimination. Argentina has reported no cases since 2013 and other countries, such as Costa Rica and El Salvador, reported less than ten cases per year. Ten Central American and Caribbean countries (Belize, Costa Rica, Dominican Republic, El Salvador, Guatemala, Haiti, Honduras, Mexico, Nicaragua, and Panama) have joined the regional initiative towards Malaria Elimination in Mesoamerica and Hispaniola (EMMIE) in 2020 [2] as well as the Malaria Certification in the Americas Region by 2025 supported by the Global Fund for AIDS, Tuberculosis and Malaria (GFATM) [2, 3]. In addition, countries in South America, such as Argentina and Paraguay, are also supporting efforts toward malaria elimination [1, 4].

The 21 countries considered at risk for malaria transmission in the Americas have adopted policies of malaria vector control using indoor residual spraying (IRS) and/or long lasting insecticide–treated mosquito nets (LLINs) in specific areas of continuous transmission [1]. These measures primarily affect endophagic and endophilic behaviour leaving mosquitoes with exophagic and exophilic behaviour with ample opportunity to bite without coming into contact with treated surfaces. Transmission caused by mosquito bites outdoors and before a community retires to sleep is known as ‘residual transmission’ [5]. In Colombia between 40 and 47 *Anopheles* species have been found [6], from which ten have been incriminated as malaria vectors: *Anophelesdarlingi, Anophelesalbimanus, Anophelesnuneztovari s.l, Anophelesneivai, Anopheles punctimacula, Anophelespseudopunctipennis, Anophelespholidotus (*as *Anopheleslepidotus)* [7], *Anophelescalderoni* [8], *Anophelesrangeli*, and *Anophelesoswaldoi* [9]. All vectors exhibit a tendency to bite more outdoors than indoors, and rest outdoors [8, 10–13]. This behaviour has been considered a major obstacle for malaria control in many countries of the Americas in which IRS is the main control measure [14].

Currently, there is a need to adopt additional strategies that will impact the *Anopheles* species with partially exophagic and exophilic habits in order to reduce the incidence of malaria in Latin America countries and to reach the pre-elimination and elimination phases, and thus, treatment of potential larval habitats could be considered an additional strategy [15].

The use of larvicides and biological control has been shown to be effective for the control of malaria globally [16]. The use of larvivorous nematode species, such as *Romanomermis culicivorax,* bacterial preparations based on *Bacillus thuringiensis* variety *israelensis* (*Bti*) and *Bacillus sphaericus* (*Bsph*) have been shown to be highly effective against African and Latin America malaria vectors, reducing larvae densities by up to 90 %, and showed even reduction in malaria prevalence in schoolchildren [17–21]. An alternative is the introduction of larvivorous fish of the species *Oreochromis spirulus*, which has been shown that to reduce *Anopheles* spp. larval density. However, more studies to examine effects on malaria in humans and on the entomological inoculation rate or at least the density of adult vector mosquitoes will be necessary [22, 23].

The most important malaria control measures are directed towards mosquitoes inside homes. However, in Latin America a great proportion of human-vector contact occurs outdoors [14, 24]. The objective of this study was to increase the knowledge regarding larval habitats in endemic populations in order to determine the feasibility of treating larval habitats, to diminish human-vector contact and contribute towards efforts for malaria elimination in the region.

## Methods

### Study area

**Fig. 1:**
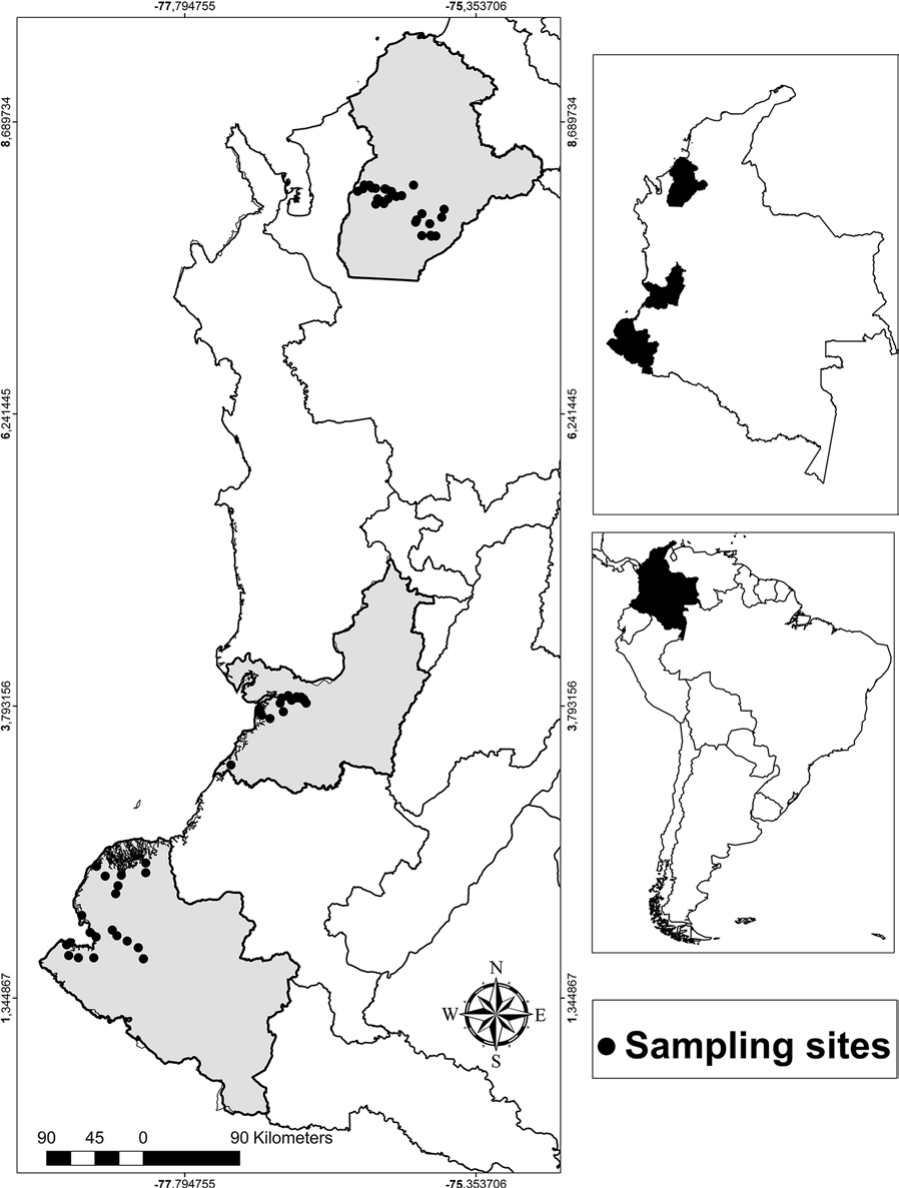
Colombian localities included in the study. *Black dots* indicate larval habitats sampled in the three states, shown in *grey* in the *left panel*. These states are shown in *black* in the map of Colombia on the *upper right panel*, and Colombia’s location is shown in *black* in the South America map on the *lower right panel*

The study took place in three of the states with the highest malaria transmission in Colombia: Córdoba (northwestern region of the country), Nariño and Valle del Cauca (both in the western region on the Pacific coast). In general, all areas follow an endemic-epidemic [25] and perennial pattern of transmission [26]. Between them a distinctive predominance of *Plasmodium* species is found. In Córdoba, 70 % of malaria infections are caused by *Plasmodium vivax* and 30 % by *Plasmodium falciparum*, while on the Pacific coast, the *P. vivax/P. falciparum* ratio is reversed with *P. falciparum* as the predominant parasite [25]. In total, 70 localities were selected for a cross-sectional study: 27 in Cordoba, 21 in Valle del Cauca and 22 in Nariño. The selection criteria included localities with high malaria incidence, easy access by land or river, and safety (Fig. 1).

The localities selected in Córdoba are in an area with northern latitudes between 07°53′53″ and 08°15′41″, and western longitudes between 75°25′30″ and 76°08′49″. Most of the localities are hilly. The annual average rainfall is between 1525 and 2333 mm and the annual average temperature between 26.4 and 28 °C depending on the altitude. The most important economic activities are based on livestock, agriculture (mainly maize, cassava, sorghum, rice, banana crops), forestry, and mining of gold and silver [27]. The localities selected in Nariño and Valle del Cauca are located in an area with northern latitudes between 01°47′55″ and 03°53′36″ and western longitudes between 77°04′11″ and 78°48′56″. Most of the localities are flat to slightly undulating. The annual average rainfall is between 2,191 and 6,980 mm and the annual average temperature between 25.8 and 27 °C. The most important economic activities are based on fishing, with some mining and agriculture (mainly banana, blackberry, cocoa, other local fruits) [27].

### Larval habitat characterization

Larval specimen collections were carried out in each of the 70 localities with data collection between May 2011 and November 2012. Each locality was visited for 1 week each during which all potential larval habitats present within a 1-km radius of the houses were sampled once for anopheline larvae between the hours of 08:00 and 12:00. The larval habitats were characterized and classified according to their distance to the nearest house, stability (temporary or permanent habitats), presence of vegetation, water flow (stagnant water or with movement), water clarity (clear or turbid), use (drainage, swimming pool, animal, domestic, none) and type defined as excavation site (a hole made by removing material), fishpond (pond or artificial lake used for fish farming), lagoon (a body of fresh water of considerable size, surrounded by land), stream (a body of water flowing in a channel, as a brook), puddle (a small pool of water, as of rainwater on the ground), ditch (a long, narrow channel dug in the ground, such as for drainage or irrigation, trench), bromeliad (epiphytic tropical American plants, having long, stiff leaves and showy flowers) or other.

### Larval sampling

In each potential habitat larval sampling was done using the standard dipping method with a 400-ml ladle with ten samples per sq m [28]. Collected larvae were maintained for linked rearing and the larval and pupal skins kept for taxonomic determination [29]. A portion of the late instars were immediately preserved in 70 % ethanol and taken to the Medical Entomology Laboratory of the Instituto Nacional de Salud of Colombia in Bogota. Species of mosquito larvae were determined using the most recent Colombian morphological *Anopheles* key [30].

### Data analysis

In order to analyse the stability of the different types of positive larval habitats for *Anopheles* larvae by state, contrast homogeneity was performed on qualitative variables using RWizard 1.0 (R 3.1.2, The R Project for Statistical Computing). A multiple correspondence analysis (MCA) was used (using R software version 3.2.0, packages ade4 and FactoMineR) to describe the main characteristics associated with each *Anopheles* species larval habitats. The categorical variables included were: stability, type, presence of vegetation, water flow, clarity of water, and use. The Fager’s affinity index (IFM) [31] was calculated to determine the association between the different anopheline species occurring in the same breeding site according to the following expression: IFM: J/√N_A_N_B_ − 1/2√N_B_, where J is the number of co-occurrences, N_A_ is the total number of occurrences of species A alone, N_B_ is the total number of occurrences of species B alone and species are chosen such that N_A_ ≤ N_B_. The resulting value provides a quantitative measure of species association. A value ≥0.5 is indicative of affinity. To analyse the interactions between *Anopheles* species and larval habitats, networks of interaction were constructed using the R statistical software (R Development Core Team 2007). In this model, the abundance and diversity of species by larval habitat were evaluated. Shannon’s diversity index (H′) was used to characterize species diversity in each state to show the abundance and evenness of the species present in the different larval habitats according to the following expression: H′: Σ((Pi) × Ln(Pi)), where Pi is number of individuals of species/total number of samples [32]. Analysis of variance (ANOVA) was used to evaluated the distance between the inspected and positive sites and the nearest houses for each area of study.

## Results

A total of 1,116 potential larval habitats were inspected in the three states, 17.5 % (195) of which were found positive for different *Anopheles* species. The state with the highest number of potential larval habitat sites inspected was Valle del Cauca (700), followed by Nariño (242) and then Córdoba (174). Cordoba was the state with the highest proportion of positive larval habitats (37.4 %) (Table 1).

**Table 1:** Larval habitats inspected, larvae presence (positive for larvae) and number of larvae of the different *Anopheles* species found in each study site

State	Number of larval habitats sampled	Positive	% Positive	Larvae (n)	% Larval habitats positive for *Anopheles* species (number of larval habitats positive for each specie/total positive habitats)						
					*An. albimanus*	*An. nuneztovari s.l.*	*An. darlingi*	*An. triannulatus*	*An. pseudopunctipennis*	*An. calderoni*	*An. neomaculipalpus*
Cordoba (a)	174	65	37.4	439	17 (11/62)	55 (36/62)	3 (2/62)	55 (36/62)	2 (1/62)		8 (5/62)
Nariño (b)	242	42	17.4	333	81 (34/42)					29 (12/42)	2 (1/42)
Valle del Cauca (c)	700	88	12.6	911	1 (1/88)	88 (77/88)			17 (15/88)		8 (7/88)
Overall	1116	195	17.5	1683							

The larval habitats were sampled once in different months of the year. Sampling months: (a) April–November 2012, (b) May–September 2011 and April–September 2012, (c) April–September 2012

A total of 1683 larvae were identified belonging to seven species. These species were *An. albimanus,An. calderoni*, *An. darlingi*, *An. neomaculipalpus*, *An. nuneztovari s.l.*, *An. pseudopunctipennis* and *An. triannulatus* (Table 1). Cordoba was the state with the highest diversity of *Anopheles* species (six) (Shannon diversity index H′: 1.063). Although Valle del Cauca had four species, one more than Nariño, the Shannon diversity index was lowest as one species, *An. nuneztovaris.l.,* predominated. The breeding site types with the highest Shannon diversity index were lagoons (H′: 1.079) and fishponds (H′: 1.009) in Cordoba, excavation sites in Nariño (H′: 0.620) and puddles in Valle del Cauca (H′: 0.764). In Cordoba, the fishponds had a higher number of *Anopheles* species than lagoons; the diversity in fishponds was lower because *An. triannulatus* and *An. nuneztovari s.l.* were predominant in this type of habitat (Table 2).

**Table 2:** Shannon diversity indices (H′) of *Anopheles* species in different types of larval habitats in each state studied

State (no. larvae found)	Type of larval habitat	Species	No. larvae	Proportion (Pi)^a^	Loge Pi	Pi Loge Pi	H′ by type of larval habitat	H′ by state
Córdoba (439)	Excavation site	*An.nuneztovari s.l.*	5	0.119	−2.128	−0.253	0.583	1.063
		*An.darlingi*	1	0.024	−3.738	−0.089		
		*An. triannulatus*	35	0.833	−0.182	−0.152		
		*An. neomaculipalpus*	1	0.024	−3.738	−0.089		
	Fishpond	*An. albimanus*	22	0.071	−2.639	−0.189	1.009	
		*An. nuneztovari s.l.*	125	0.406	−0.902	−0.366		
		*An. darlingi*	4	0.013	−4.344	−0.056		
		*An. triannulatus*	154	0.500	−0.693	−0.347		
		*An. pseudopunctipennis*	2	0.006	−5.037	−0.033		
		*An. neomaculipalpus*	1	0.003	−5.730	−0.019		
	Lagoon	*An. albimanus*	2	0.286	−1.253	−0.358	1.079	
		*An. nuneztovari s.l.*	2	0.286	−1.253	−0.358		
		*An. triannulatus*	3	0.429	−0.847	−0.363		
	Stream	*An. nuneztovari s.l.*	3	0.750	−0.288	−0.216	0.562	
		*An. triannulatus*	1	0.250	−1.386	−0.347		
	Puddle	*An. albimanus*	8	0.118	−2.140	−0.252	0.894	
		*An. nuneztovari s.l.*	49	0.721	−0.328	−0.236		
		*An. triannulatus*	6	0.088	−2.428	−0.214		
		*An. neomaculipalpus*	5	0.074	−2.610	−0.192		
	Ditch	*An. albimanus*	3	1.000	0.000	0.000	0.000	
	Other	*An. nuneztovari s.l.*	1	0.143	−1.946	−0.278	0.410	
		*An. triannulatus*	6	0.857	−0.154	−0.132		
Nariño (333)	Excavation site	*An. albimanus*	95	0.688	−0.373	−0.257	0.620	0.432
		*An. calderoni*	43	0.312	−1.166	−0.363		
	Lagoon	*An. neomaculipalpus*	1	1.000	0.000	0.000	0.000	
	Puddle	*An. albimanus*	128	0.985	−0.016	−0.015	0.079	
		*An. calderoni*	2	0.015	−4.174	−0.064		
	Ditch	*An. albimanus*	22	1.000	0.000	0.000	0.000	
	Other	*An. albimanus*	39	0.929	−0.074	−0.069	0.257	
		*An. calderoni*	3	0.071	−2.639	−0.189		
Valle del Cauca (911)	Excavation site	*An. nuneztovari s.l.*	98	0.970	−0.030	−0.029	0.134	0.399
		*An. pseudopuncti* *pennis*	3	0.030	−3.517	−0.104		
	Fishpond	*An. nuneztovari s.l.*	431	0.995	−0.005	−0.005	0.029	
		*An. pseudopuncti*-*pennis*	2	0.005	−5.378	−0.025		
	Lagoon	*An. neomaculipalpus*	4	1.000	0.000	0.000	0.000	
	Stream	*An. nuneztovari s.l.*	80	0.889	−0.118	−0.105	0.349	
		*An. pseudopuncti* *pennis*	10	0.111	−2.197	−0.244		
	Puddle	*An. albimanus*	1	0.004	−5.537	−0.022	0.764	
		*An. nuneztovari s.l.*	189	0.744	−0.296	−0.220		
		*An. pseudopuncti* *pennis*	31	0.122	−2.103	−0.257		
		*An. neomaculipalpus*	33	0.130	−2.041	−0.265		
	Ditch	*An. pseudopuncti* *pennis*	4	1.000	0.000	0.000	0.000	
	Other	*An. nuneztovari s.l.*	22	0.880	−0.128	−0.112	0.367	
		*An. neomaculipalpus*	3	0.120	−2.120	−0.254		

The larval habitats were sampled once in different months of the year

^a^Proportion: number of individuals of species/total number collected in each type of larval habitat

**Fig. 2:**
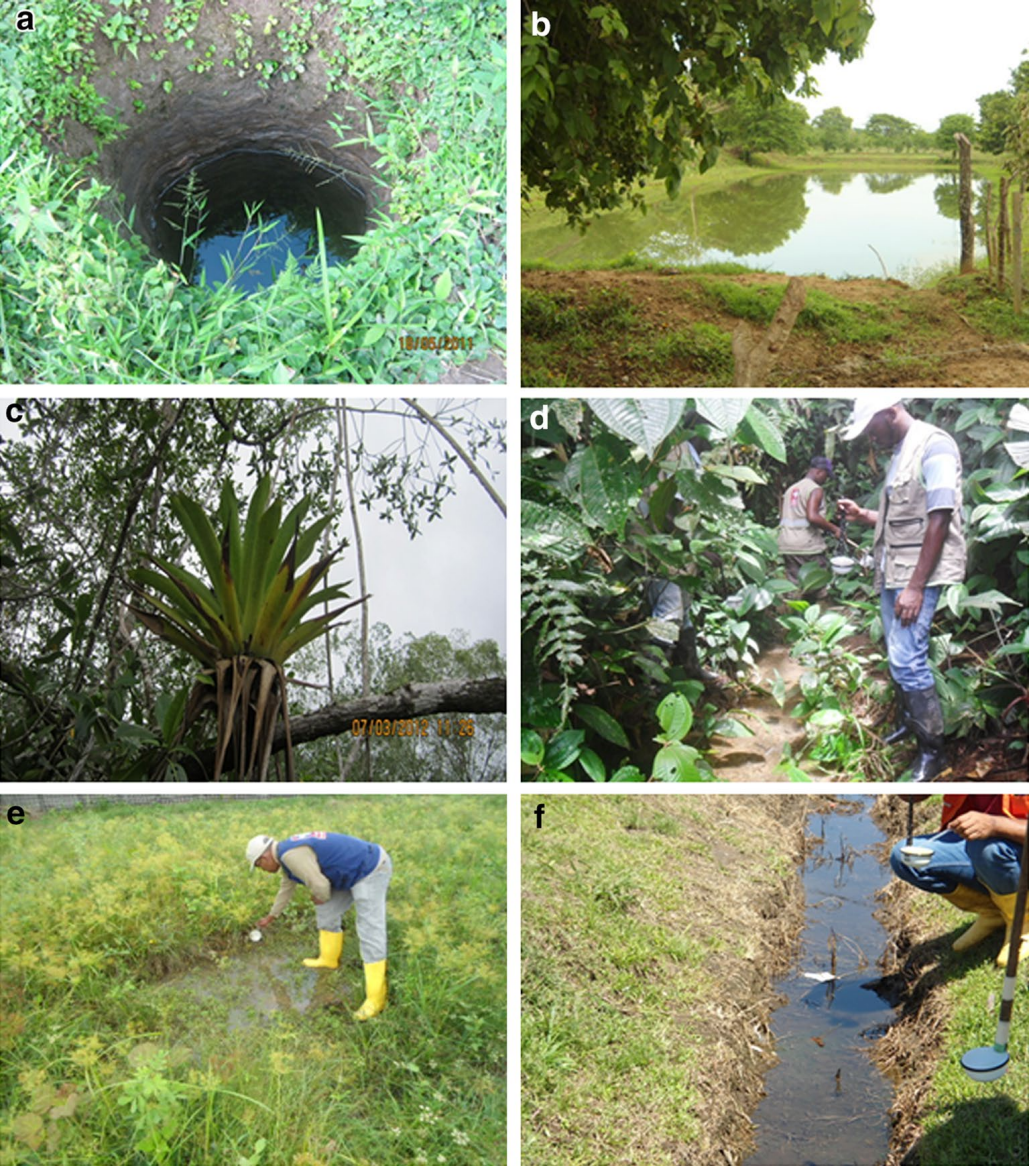
Larval habitats inspected for *Anopheles* larvae in Cordoba, Valle del Cauca and Nariño between 2011 and 2012. **a** Excavation sites, **b** fishponds, **c** bromeliads, **d** streams, **e** puddles, **f** ditches

Table 3 shows the characteristics of mosquito larval habitats such as vegetation presence, water flow, water clarity, stability, habitat types, and uses. The most common larval habitat types found were excavation sites, fishponds, bromeliads, streams, pools, ditches, and lagoons (Fig. 2a–f).

**Table 3:** Characteristics of larval habitats for *Anopheles* species in Cordoba, Valle del Cauca and Nariño

Characteristic of larval habitats		Cordoba				Nariño		Valle del Cauca		
		% ABM n = 15	% NTV n = 41	% TRI n = 37	% NEO n = 5	% ABM n = 34	% CAL n = 12	% NTV n = 77	% PPP n = 15	% NEO n = 7
Stability	Temporary	27.3	22.2	5.6	60	30.3	16.7	28.6	33.3	28.6
	Permanent	77.7	77.8	94.4	40	69.7	83.3	71.4	66.7	71.4
Type	Excavation site	–	8.6	11.4	20	69.7	75	16.9	6.7	–
	Fishpond	45.5	60	71.4	20	–	–	27.3	13.3	–
	Lagoon	9.1	2.9	2.9	–	–	–	–	–	14.3
	Stream	–	2.9	2.9	–	–	–	6.5	26.7	–
	Puddle	36.4	22.9	5.7	60	24.2	16.7	46.8	46.7	71.4
	Ditch	9.1	–	–	–	3	–	–	6.7	–
	Other	–	2.9	5.7	–	3	8.3	2.6	–	14.3
Vegetation	Present	100	100	100	100	95.9	88.2	96.6	100	92.9
	Absent	–	–	–	–	4.1	11.8	3.4	–	7.1
Water flow	Stagnant water	100	97.2	94.4	100	97.1	100	93.5	73.3	100
	With movement	–	2.8	5.6	–	2.9	–	6.5	26.7	–
Water clarity	Clear	18.2	11.1	11.4	–	72.7	75	51.9	73.3	57.1
	Turbid	81.8	88.9	88.6	100	27.3	25	48.1	26.7	42.9
Use	Drainage	16.7	–	3.4	–	3.2	–	–	6.7	–
	Swimming pool	–	–	–	–	–	–	1.3	–	14.3
	Animal	83.3	90.9	86.2	50	–	–	27.3	13.3	
	Domestic	–	4.5	3.4	–	35.5	45.5	–	–	–
	None	–	4.5	6.9	50	61.3	54.5	71.4	80	85.7

ABM: *An. albimanus*, NTV: *An. nuneztovari s.l.*, DAR: *An. darlingi*, TRI: *An. triannulatus*, PPP: *An. pseudopunctipennis*, NEO: *An. neomaculipalpus*, CAL: *An. calderoni*

The larval habitats were sampled once in different months of the year

**Fig. 3:**
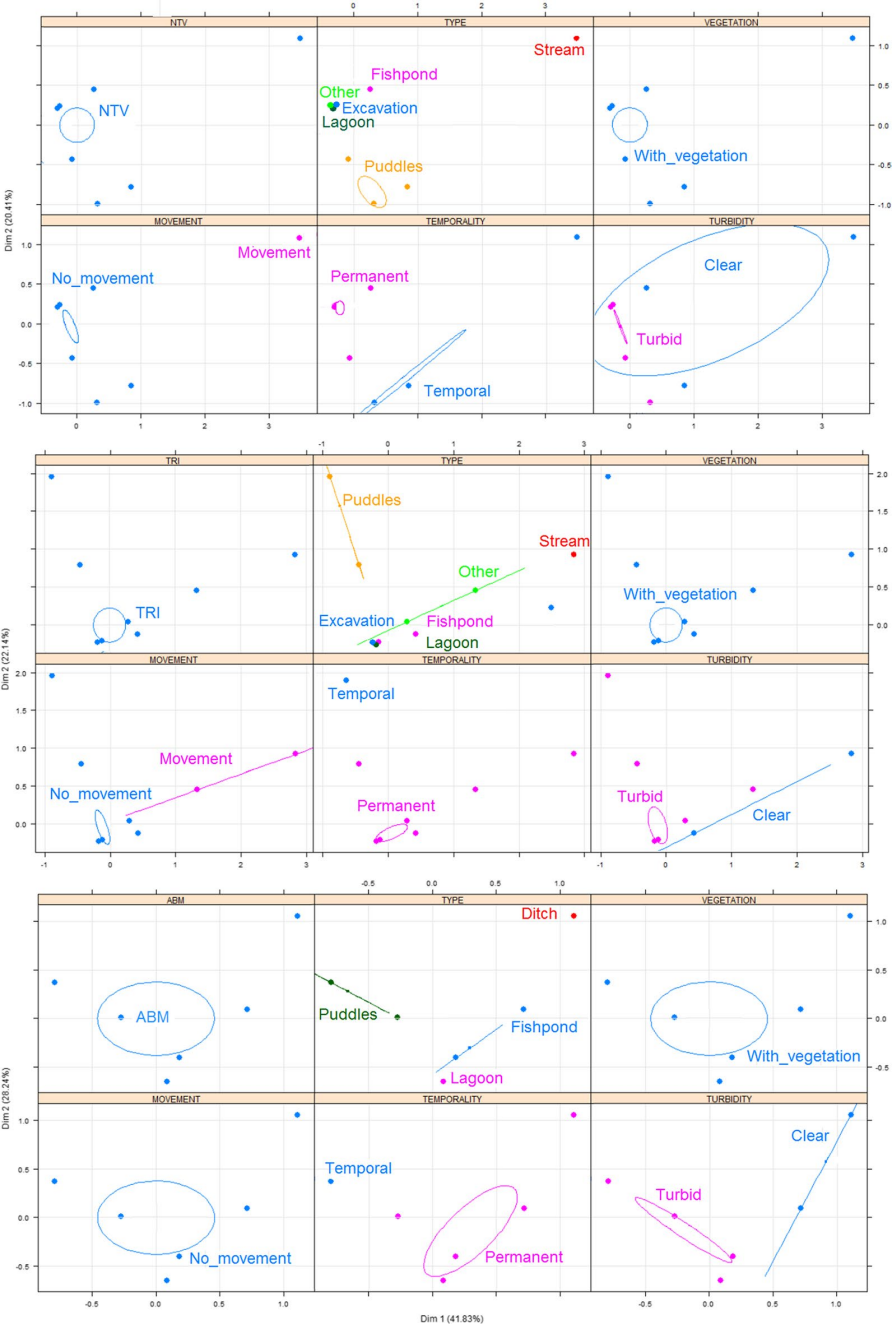
Multiple correspondence analysis (MCA) with the main characteristics associated with *Anopheles* larval habitats in Cordoba. **a** *An. nuneztovari* *s.l*., **b** *An. triannulatus*, **c** *An. albimanus*. *The larval habitats were sampled once in different months of the year

**Fig. 4:**
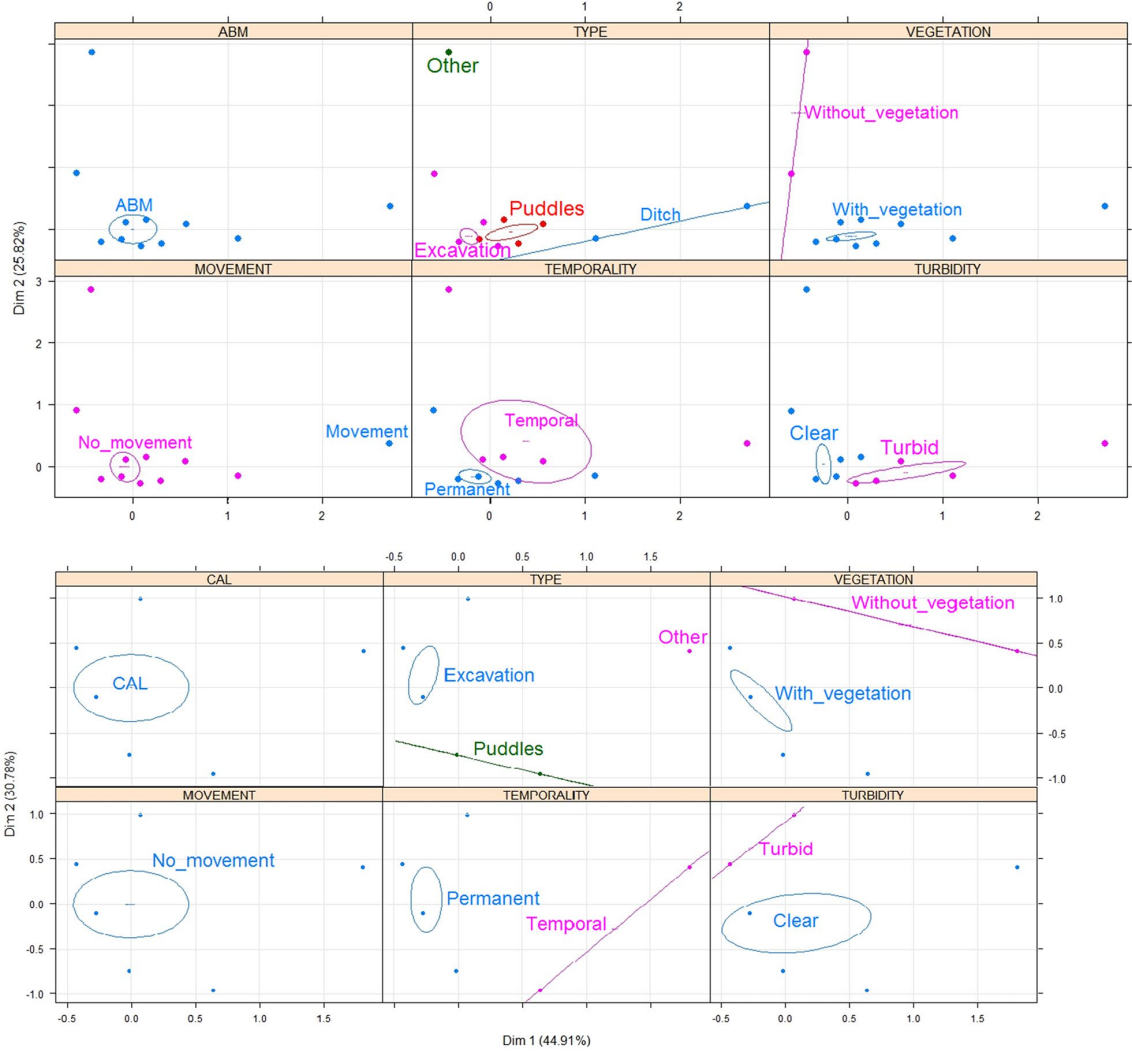
Multiple correspondence analysis (MCA) with the main characteristics associated with *Anopheles* larval habitats in Nariño. *The larval habitats were sampled once in different months of the year. **a** *An. albimanus*, **b** *An. calderoni*

**Fig. 5:**
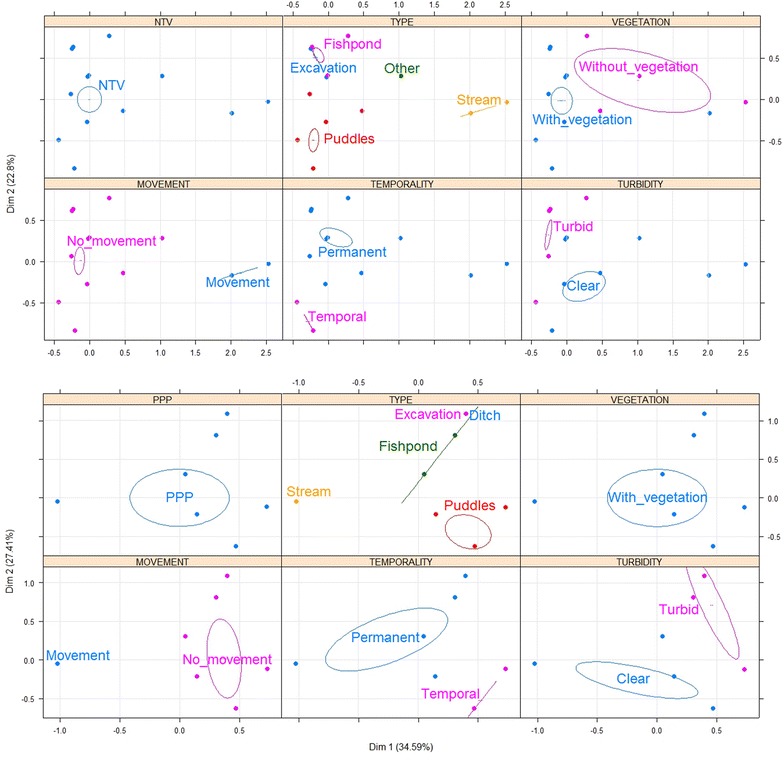
Multiple correspondence analysis (MCA) with the main characteristics associated with *Anopheles* larval habitats in Valle del Cauca. *The larval habitats were sampled once in different months of the year. **a** *An. nuneztovari* *s.l*., **b** *An. pseudopunctipennis*

In Cordoba, *An. nuneztovari s.l.* larvae were found in larval habitats characterized by having vegetation, stagnant water and clear water (Fig. 3a). *Anopheles triannulatus* were found in fishponds used for commercial rearing of fish, the majority of which were permanent; these larval habitats had vegetation, turbid and stagnant water (Fig. 3b). Larvae of *An. albimanus* were found in larval habitats that were permanent, characterized by having vegetation, turbid and stagnant water (Fig. 3c). In Nariño, *An. albimanus* were found in puddles and excavation sites used for domestic activities, such as washing dishes, cleaning floors and even cooking, the majority of which were temporal. These larval habitats had stagnant water and vegetation (Fig. 4a). Larvae of *An. calderoni* were present in excavation sites used for domestic activities. These larval habitats were characterized by being permanent, having vegetation and stagnant water (Fig. 4b). In Valle del Cauca, the presence of *An. nuneztovari s.l.* was associated with permanent larval habitats with stagnant water and vegetation (Fig. 5a). Finally, *An. pseudopunctipennis* were found in fishponds and puddles without use, the majority of which were permanent. These larval habitats had stagnant water and vegetation (Fig. 5b). The type and use of larval habitats showed association in most cases, i.e., fishponds were used for rearing fish, puddles had no use, excavations were mainly used for domestic purposes, etc.; therefore, the MCA considered the variable type only to avoid redundancy for autocorrelation.

**Fig. 6:**
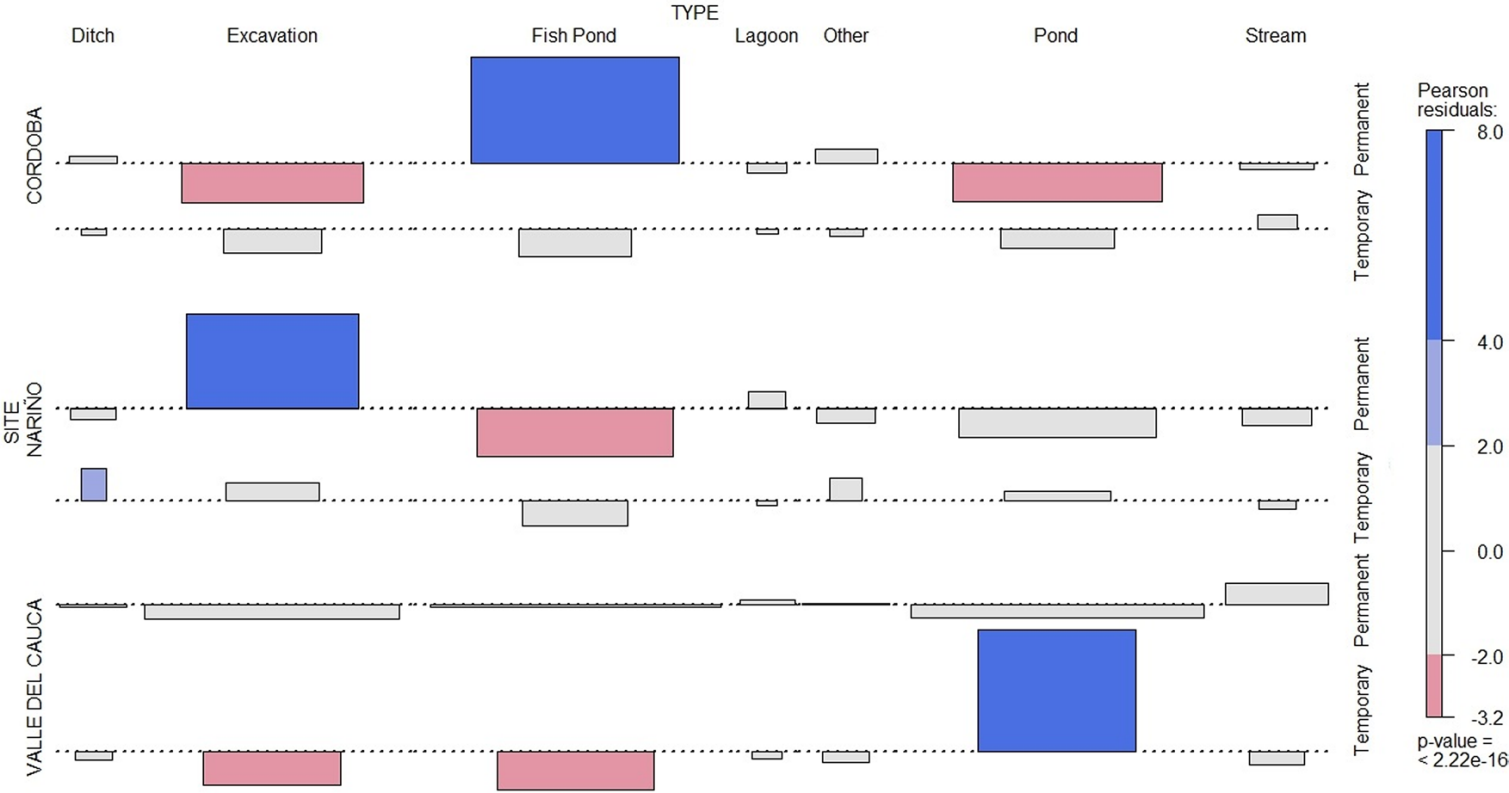
Association between type of larval habitat in which *Anopheles* larvae were found and their temporality. Qualitative variable homology analysis (Pearson’s Chi squared test) showing in *blue boxes* positive and significant association (p < 0.05). *Pink boxes* negative and significant association, *grey boxes* no association (n = 173). *The larval habitats were sampled once in different months of the year

The analysis of contrast homogeneity for qualitative variables showed significant differences regarding the stability of the larval habitats; fishponds in Cordoba and excavation sites in Nariño were associated with permanent larval habitats, while in Valle del Cauca the puddles were associated with being temporary compared to other types of larval habitats inspected (Fig. 6).

**Fig. 7:**
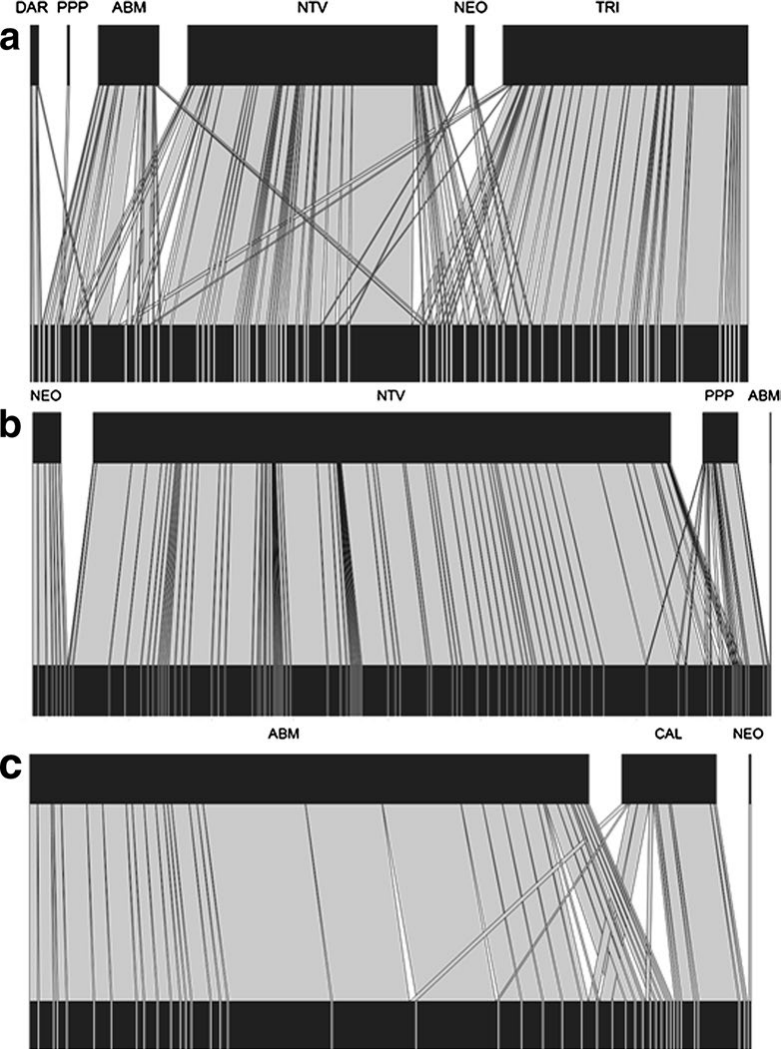
Network interactions between species of *Anopheles* and larval habitats. *The larval habitats were sampled once in different months of the year. **a** Cordoba, **b** Valle del Cauca, **c** Nariño. The *black bands* above the figures show the abundance of each *Anopheles* species, and *black bands* below show every larval habitat. The *perpendicular lines* show the presence of each species in site. ABM: *An. albimanus*, NTV: *An. nuneztovari s.l.*, DAR: *An. darlingi*, TRI: *An. triannulatus*, PPP: *An. pseudopunctipennis*, NEO: *An. neomaculipalpus*, CAL: *An. calderoni*

Results from the interaction network analysis showed that in Cordoba *An.triannulatus, An. neomaculipalpus, An. nuneztovari s.l.*, and *An. albimanus* shared some larval habitats and that the highest abundance was found for *An. nuneztovari s.l.* and *An. triannulatus* (Fig. 7a). In Nariño, *An. albimanus* was the species with the highest abundance in the localities inspected and *An. calderoni* shared larval habitats with *An. albimanus* (Fig. 7b). In Valle del Cauca, *An. nuneztovari s.l.* showed the highest abundance and shared larval habitats with *An. pseudopunctipennis* (Fig. 7c).

Only *An. nuneztovari s.l.* and *An. neomaculipalpus* showed a significant IFM (0.67) and shared larval habitats in Cordoba state. The other species, although also sharing larval habitats, showed an association that was not significant (Table 4). *Anopheles neomaculipalpus* in Nariño and *An. albimanus* in Valle del Cauca did not share any larval habitats with others species.

**Table 4:** Fager’s affinity index between *Anopheles* species in the study area during 2011–2012

Córdoba	ABM	DAR	NEO	NTV	PPP	TRI
ABM	**–**	−0.15	−0.15	0.27	0.15	0.07
DAR		**–**	−0.22	−0.08	−0.35	−0.08
NEO			–	0.67	−0.22	0.14
NTV				**–**	0.08	0.25
PPP					**–**	−0.08
TRI						**–**
Nariño	ABM	CAL	NEO			
ABM	**–**	0.16	−0.09			
CAL		**–**	−0.14			
NEO			**–**			
Valle del Cauca	ABM	NEO	NTV	PPP		
ABM	**–**	−0.19	−0.06	−0.13		
NEO		**–**	−0.01	−0.13		
NTV			**–**	0.27		
PPP				**–**		

ABM: *An. albimanus*, NTV: *An. nuneztovari s.l.*, DAR: *An. darlingi*, TRI: *An. triannulatus*, PPP: *An. pseudopunctipennis*, NEO: *An. neomaculipalpus*, CAL: *An. calderoni*

The larval habitats were sampled once in different months of the year. Indexes > 0.5 means association between the different anopheline species occurring in the same larval habitat [31]

Compared to *Anopheles*-negative larval habitats, *Anopheles*-positive larval habitats were further from the houses, although this difference was only significant in Valle del Cauca (Table 5). A significant difference was also found between states regarding the distance between houses and positive larval habitats (F = 30.8; p < 0.001); the closest proximity between houses and larval habitats was in Nariño (mean distance = 22.7 m), followed by those in Cordoba (57.2 m), and the greatest mean distance was in Valle del Cauca (205.7 m).

**Table 5:** Average distance between negative and positive larval habitats and the nearest house in the states of Cordoba, Nariño and Valle del Cauca (2011–2012)

SITE (n)	Sampled larval habitats	Positive larval habitats	Negative larval habitats	df	F	*p* *value*
	Mean (SD)	Mean (SD)	Mean (SD)			
Córdoba (172)	56.3 (56.3)	57.2 (52.4)	55.8 (58.7)	1; 170	0.25	0.874
Nariño (221)	20.12 (32.7)	22.7 (35.0)	19.5 (32.3)	1; 219	0.31	0.578
Valle del Cauca (699)	143.2 (183.7)	205.7 (21.9)	136.8 (179.6)	1; 697	5.93	0.015**

ANOVA (α = 0.05)

*SD* standard deviation, *df* degrees of freedom

The larval habitats were sampled once in different months of the year

** Significant p < 0.05

## Discussion

Larval habitats found in the most malaria-endemic regions in Colombia were characterized in a cross-sectional study, regarding their physical description, association between the different anopheline species, species diversity and distance between positive sites and the nearest houses for each area of study, in order to determine the feasibility of treating larval habitats to diminish human vector contact and contribute toward efforts for malaria elimination in the region. The main larval habitats found were permanent and human-made, such a fishponds and excavations for domestic use, which can be treated. Fishponds were the most abundant and positive for *An. nuneztovari s.l.* larvae in the northwest (state of Cordoba), whereas wells were the main larval habitats for *An. albimanus* in the southwest in Nariño. In contrast, temporary puddles were the main larval habitats for *An. nuneztovari s.l.* in Valle del Cauca, which are likely rain-dependent. The WHO recognizes that the treatment of potential larval habitats can be considered an additional strategy for the control of malaria in areas where these are few, identifiable and easy to access [15], which is the case for both permanent human-made types of larval habitats found in this study: fishponds and wells or excavation sites. These larval habitats could be targeted for treatment according to WHO guidelines [15].

The presence of fishponds has been recognized as a serious threat for malaria transmission in other Latin America countries, such as Peru [33] and Brazil [34], where the presence and number of fishponds has been associated with an increase in malaria cases. In those countries, *An. darlingi*, the most important neotropical malaria vector, seems to have adapted well to fishponds despite predation by fish juveniles [35, 36]. In the Iquitos-Nauta Road in the Peruvian Amazons, there was a higher number of self-reported malaria episodes in households located closer to fishponds, the most commonly positive larval habitats in this area [26]. In the same area, Maheu-Giroux et al. [37] found evidence of fishpond density as a major risk factor for malaria transmission.

Wells or excavation sites are common in rural Nariño, in the southwest of the country. Nearly all houses have one as a source of water for domestic purposes. The main malaria vector species in this area, *An. albimanus* and *An. calderoni*, were associated with this type of larval habitat. Wells have been recognized as suitable larval habitats for mosquitoes, including as a refuge during the dry season for *Anopheles*, *Aedes* and *Culex* species [38], and treatment using larvivorous fish in wells has been associated with a reduction in malaria transmission [39].

One of the limitations of this study is related to its design. This was a cross sectional study where every larval habitat was sampled only once. This information provided a general overview of the characteristics of larval habitats present in a great variety of malaria-endemic localities. However data collected are insufficient neither to carry out a more specialized analysis nor to estimate possible associations between larval habitats, seasonality nor climate.

Given that the most common larval habitats of the main malaria vector species in two sites were human-made and permanent water bodies (fishponds and wells), which people use for economic activities (fish rearing) or provision of water for domestic purposes, these habitats cannot be eliminated but could and should be treated. Different possibilities could be explored avoiding any harm to the reared fish or the humans who may consume the water, particularly that from wells or excavation sites.

*Anopheles albimanus* larvae were found in the three states, principally associated to larval habitats with standing water and the presence of vegetation. This species was associated with permanent larval habitats with turbid water in Córdoba, as in other parts of Latin America [24, 30–42]. The results showed that *An. albimanus* exploits a wide variety of larval habitats, including fishponds, lagoon, puddles, ditches, and excavation sites [41–48], which would make control of this species difficult and could favour high densities of adult mosquitoes in its distribution range. According to the results of the MCA, association of *An. albimanus* with certain types of larval habitats was only statistically significant in Nariño where this species was found mainly associated with puddles without any use and excavation sites used for domestic purposes. In this study, *An. albimanus* was found sharing larval habitats with *An. calderoni*, *An. neomaculipalpus, An. pseudopunctipennis, An. nuneztovari s.l., An. darlingi*, and *An. triannulatus*, which indicates its plasticity [42–49].

Little was known regarding the characteristics of *An. calderoni* larval habitats. According to Wilkerson [50], larvae of this species are found mainly in small streams, small irrigation canals and swamps, mostly in dense emergent vegetation. In this study, *An. calderoni* was associated mainly with human-made wells or excavation sites with standing water used for domestic activities.

Larvae of *An. nuneztovari s.l.* were present more often in habitats with clear stagnant water characterized by having vegetation [51, 52]. This species was related to permanent habitats in Córdoba, but to temporary ones in Valle del Cauca, showing that *An. nuneztovari s.l.* can be present in larval habitats regardless of their temporality [52–54]. Although *An. nuneztovari s.l.* was collected with other species, such as *An. albimanus*, *An. darlingi*, *An. pseudopunctipennis*, *An. triannulatus*, and *An. neomaculipalpus* [36, 51, 55–58], the affinity index was significant only with *An. neomaculipalpus* in Cordoba. In this study, *An. nuneztovari s.l.* was collected in excavation sites, fishponds, lagoons, streams, puddles, and ditches but the MCA results did not show an association between the species and any particular larval habitat type. This may reflect the availability of possible sites more than a particular species preference for any larval habitat type.

A clear association was observed between *An. triannulatus* larvae and standing water in fishponds surrounded by vegetation. These results contradict those found in Amazonian Brazil [59], Chiapas in Mexico [45] and Perú [56] where this species showed a more generalist habitat colonization and exploited larval habitats such as lakes, streams with slow currents, slow-moving rivers, large ponds, mining excavation sites, ditches, or marshes. This is the first time this species is found associated with standing and muddy water. *Anopheles triannulatus* was found sharing larval habitats with *An. albimanus*, *An. darlingi*, *An. neomaculipalpus*, *An. nuneztovari s.l.*, and *An. pseudopunctipennis* [49, 51, 53, 54, 57–61]. However, the affinity index for *An. triannulatus* was not significant for any combination of species.

The presence of larval habitats near houses has been found to be associated with abundance of mosquito larvae [62] and malaria transmission risk [63], mainly in Africa. However, in this study, *Anopheles*-positive larval habitats were found further from the nearest house compared to *Anopheles*-negative larval habitats. This difference was only significant for the temporary puddles in Valle del Cauca in which *Anopheles*-negative larval habitats were significantly closer to houses compared to *Anopheles*-positive larval habitats. It is important these results be considered by the malaria programme since potential larval habitats closer to houses might be easier to treat, whereas more distant ones might remain untreated.

## Conclusions

This study of larval habitats provides information relevant to malaria programmes. In the context of Latin America malaria control or elimination programmes, other control measures are necessary beyond indoor targeting of adult mosquitoes, such as LLINs or IRS. Treatment of larval habitats may be an appropriate complementary option since the main larval habitats found were human-made (permanent fishponds and wells or excavation sites), well defined, and feasible to control. Local evaluation of larval control strategies should be implemented and evaluated.

## Abbreviations

*Bsph*: *Bacillus sphaericus*
*Bti*: *Bacillus thurigiensis* variety israelensis
EMMIE: malaria elimination in Mesoamerica and Hispaniola
H′: Shannon diversity index
IGAC: Instituto Geográfico Agustin Codazzi
IRS: indoor residual spraying
LLINs: long-lasting insecticide-rreated mosquito nets
MCA: multiple correspondence analysis
WHO: World Health Organization
